# Supplementary material for: Interaction Between Nitric Oxide and Silicon on Leghaemoglobin and S-Nitrosothiol Levels in Soybean Nodules
Source: Biomolecules. 2024 Nov 7;14(11):1417. doi: 10.3390/biom14111417 (PMC11592487; doi:10.3390/biom14111417)
Supplement: Supplementary file 1 [file biomolecules-14-01417-s001.zip › biomolecules-3214789-supplementary.pdf]

Interaction between nitric oxide and silicon on leghaemoglobin and S-nitrosothiol levels in soybean nodules

Da-Sol Lee[†], Ashim Kumar Das[†], Nusrat Jahan Methela, Byung-Wook Yun*

Table S1. Primer lists used in the study.

Locus ID	Gene	Sequence
Glyma.19G170100	*Gmgsnor19g*_F	ATGGGAAGAGTCGGTTCTCG
	*Gmgsnor19g*_R	TTTCTCCAGAGGTGCCTTCG
Glyma09g37280	*GmNIP2-1*_F	ATCATCTTCCCCTGGCTCTCT
	*GmNIP2-1*_R	TAGCCCAGCAGAACCACTTC
GLYMA_11G121700	*GmLba*_F	AACATTCCTCAATACAGCGTTGTG
	*GmLba*_R	CCCGTGAGCTTAGGATTAG
Glyma07g36040.1	*GmFLbR-1*_F	TGTGAAATCTCTGCCTGGGA
	*GmFLbR-1*_R	GCCCCATACTGAGCCCATTT
Glyma17g04210.1	*GmFLbR-2*_F	GGGGATGGTGTGAAGCTAAC
	*GmFLbR-2*_R	GTCCAACCCAAGTTCGGCA
Glyma02g10170	*GmActin*-F	ACCCAGCAGCATGAAGATCA
	GmActin-R	CACATCTGCTGGAAGGTGCT

AT3G17240.1 (dihydrolipoamide dehydrogenase), Orthologs I (**Glyma07g36040.1**) ferric leghemoglobin reductase 1, FLbR-1

>Glyma07g36040.1 sequence type=CDS gene model=Glyma07g36040 sequence assembly version=Glyma 1.0 annotation version=1.1 JGI Gene Call confidence=high
ATGGCGATGGCAAACTTGGCTCGACGGAAGGGTTACGCCGTCGTTTTGTCGTCGAGGTCGTCGCTTTGTCTGACAAGGTGGAGGGGGTTCGCGTCCGGATCTGACGAAAACGACGTCGTGGTCATCGGCGGC
GGTCCCGGCGGCTACGTGGCGGCCATTAAAGCGGCGCAGCTGGGTCTGAAAACCACTTGCATCGAAAAACGTGGCACTCTCGGCGGTACCTGCCTCAACGTCGGATGCATCCCTTCCAAGGCACTTTTGCATT
CTTCCCACATGTATCATGAGGCTAAACATGCATTTGCCAACCATGGGGTCAAGTTTTCATCTGTCGAGGTTGATTTGCCAGCCATGATGGCCCAAAAAGATAAAGCAGTTTCTAATCTTACCAAGGGTATTGAAGG
TCTATTCAAGAAAAACAAGGTAAACTATGTCAAAGGTTATGGCAAATTAGTTTCACCATCTGAGGTCTCTGTGGACACCACTGAAGGTGGAAATACTGTTGTGAAAGGCAAGCATATTATAATTGCCACTGGCTCG
GATGTGAAATCTCTGCCTGGGATCACTATTGATGAAAAGAAAGTTGTTTCATCAACGGGAGCTCTTGCTTTGACTGAAATCCCCAAGAGACTCATAGTCATTGGGGCAGGCTACATTGGGCTGGAAATGGGCTCA
GTATGGGGCCGACTTGGCTCCGAGATAACAGTTGTTGAGTTTGCATCGGAGATTGTTCCAACCATGGATGCGGAGGTCCGGAAGCAGTTTCAGCGTTCTCTTGAGAAGCAAGGCATGAAATTCAAGCTGAAGAC
TAAGGTGGTTGGAGTTGATACTTCTGGGGATGGTGTGAAGCTAACTCTTGAACCAGCAGCTGGTGGTGATCAGACTACACTTGAAGCAGATGTTGTCCTGGTATCTGCTGGTAGGACCCCATTCACTGCCGAACT
TGGGTTGGACAAGATAGGAGTTGAAACTGACAAGATTGGACGGATTTTGGTAAATGAAAGATTTGCCACAAATGTCTCTGGTGTTTATGCAATTGGAGATGTAATTCCAGGCCCAATGTTGGCACACAAGGCGGA
AGAAGATGGTGTTGCTTGTGTTGAGTACATAGCTGGTAAGGTTGGTCATGTGGATTATGACAAAGTCCCTGGCGTTGTCTATACAATGCCCGAGGTTGCATCTGTTGGGAAGACAGAGGAGCAGGTGAAGGAAC
TTGGAGTTGAATACCGTGTTGGCAAGTTCCCATTCCTGGCTAATAGCAGAGCTAAGGCAATTGACAATGCTGAAGGACTGGTGAAGATATTGGCTGAAAAGGAGACAGACAAGATATTGGGAGTGCACATTATGG
CGCCCAATGCAGGAGAGCTTATTCATGAAGCTGCAATAGCACTACAGTATGATGCATCAAGTGAGGACATTGCACGTGTGTGCCATGCACATCCAACAATGAGCGAGGCTGTGAAAGAAGCCGCAATGGCCACT
TATGACAAGCCCATTCACATCTAA

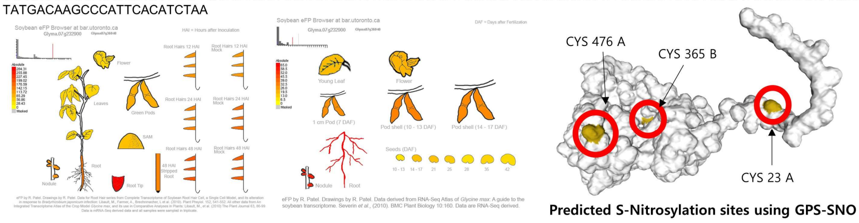

Predicted S-Nitrosylation sites using GPS-SNO 1.0

>Glyma07g36040.1 sequence type=predicted peptide gene model=Glyma07g36040 sequence assembly version=Glyma 1.0 annotation version=1.1 JGI Gene Call confidence=high
MAMANLARRKGYAVVLSSRSSL**C**LTRWRGFASGSDENDVVVIGGGPGGYVAAIKAAQLGLKTTCIEKRGTLGGTCLNVGCIPSKALLHSSHMYHEAKHAFANHGVKFSSVEVDLPAMMAQKDKAVSNLTKGIEGLFKKNKVNYVKGY
GKLVSPSEVSVDTTEGGNTVVKGKHIIIATGSDVKSLPGITIDEKKVVSSTGALALTEIPKRLIVIGAGYIGLEMGSVWGRLGSEITVVEFASEIVPTMDAEVRKQFQRSLEKQGMKFKLKTKVVGVDTSGDGVKLTLEPAAGGDQTTLEADVVL
VSAGRTPFTAELGLDKIGVETDKIGRILVNERFATNVSGVYAIGDVIPGPMLAHKAEEDGVA**C**VEYIAGKVGHVDYDKVPGVVYTMPEVASVGKTEEQVKELGVEYRVGKFPFLANSRAKAIDNAEGLVKILAEKETDKILGVHIMAPNAGE
LIHEAAIALQYDASSEDIARV**C**HAHPTMSEAVKEAAMATYDKPIHI*

Threshold	Position	Peptide	Score	Cutoff	Cluster
High	365	AEEDGVACVEYIAGK	2.565	2.443	Cluster B
Low	476	SEDIARVCHAHPTMS	1.224	0.797	Cluster A

Figure S1. Predicted S-Nitrosylation sites in the protein sequence of FLbR-1.

AT3G17240.1 (dihydrolipoamide dehydrogenase), Orthologs II (**Glyma17g04210.1**) ferric leghemoglobin reductase 2, FLbR-2

>Glyma17g04210.1 sequence type=CDS gene model=Glyma17g04210 sequence assembly version=Glyma 1.0 annotation version=1.1 JGI Gene Call confidence=high
ATGGCGATGGCAAGCCTGGCTCGACGGAAGGGTTACGCCGTCGTTTTGTCGTCGAGGTCGTCGTTTTGTCTGACAAGCTGGAGGGGATTCGCGTCCGGATCTGACGAAAACGACGTCGTCGTCATCGGCGGCGGTCCCGGCGGCTACGTGGCCGCC
ATCAAAGCCGCGCAGCTCGGTCTGAAAACCACTTGCATCGAAAAGCGTGGCACTCTCGGCGGTACCTGCCTCAACGTCGGATGCATCCCTTCCAAGGCACTTCTGCATTCTTCCCACATGTATCATGAGGCTAAACATGCATTTGCCAACCATGGG
GTCAAGTTTTCATCTGTTGAGGTTGATTTGCCAGCCATGATGGCCCAAAAAGATAAAGCAGTTTCTAATCTTACCAAGGGTATTGAAGGTCTATTCAAGAAAAACAAGGTAAACTATGTCAAAGGTTATGGCAAATTTGTTTCACCATCTGAAGTC
TCTGTGGACACCACTGAAGGTGGAAATACTGTTGTGAAAGGCAAGCATATTATAATTGCCACTGGCTCGGATGTGAAATCATTGCCTGGCGTCACTATTGATGAAAAGAAAGTCGTATCATCAACGGGGGCTCTTGCTTTGACTGAAATCCCCAAG
AGACTCGTAGTCATTGGGGCAGGCTACATTGGGCTGGAAATGGGCTCCGTATGGGGCCGTCTTGGCACCGAGATAACAGTTGTTGAATTTGCATCGGAGATTGTTCCAACCATGGATGCAGAGGTCCGAAAGCAGTTTCAGCGTTCTCTTGAGAAG
CAAGGCCTGAAATTCAAGCTGAAGACAAAGGTAGTTGGAGTTGATACTTCTGGGGATGGTGTGAAGCTAACTCTTGAACCGGCTGCTGGTGGTGATCAAACTACACTTGAAGCAGATGTTGTCCTTGTATCTGCTGGTAGGACTCCATTCACTGCT
GGACTTGGATTGGACAAGATAGGTGTTGAAACTGACAAGATTGGACGGATTTTGGTAAACGAACGATTTGCCACAAATGTCTCTGGTGTTTATGCAATTGGAGATGTAATTCCAGGCCCAATGTTGGCACACAAGGCAGAAGAAGACGGTGTTGCT
TGTGTTGAGTACATAGCTGGTAAGGTGGGCCATGTGGATTATGACAAAGTCCCCGGTGTTGTCTATACAATGCCCGAGGTTGCATCTGTTGGGAAGACAGAGGAGCAGGTGAAGGAACTTGGAGTTGAATACCGTGTTGGCAAGTTCCCGTTCATG
GCTAATAGCAGAGCTAAGGCAATTGACAATGCTGAAGGACTGGTGAAGATATTGGCTGAAAAGGAGACAGACAAGATATTGGGAGTGCACATTATGGCACCCAATGCAGGAGAGCTTATTCATGAAGCAGCAATAGCACTACAGTATGATGCATCT
AGTGAGGACATTGCACGTGTGTGCCATGCACATCCAACAATGAGCGAGGCTGTGAAAGAAGCCGCAATGGCCACTTATGACAAGCCCATTCACATTTAA

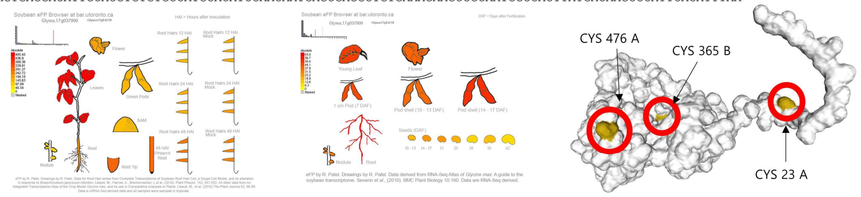

Predicted S-Nitrosylation sites using GPS-SNO 1.0

>Glyma17g04210.1 sequence type=predicted peptide gene model=Glyma17g04210 sequence assembly version=Glyma 1.0 annotation version=1.1 JGI Gene Call confidence=high
MAMASLARRKGYAVVLSSRSSF**C**LTSWRGFASGSDENDVVVIGGGPGGYVAAIKAAQLGLKTTCIEKRGTLGGTCLNVGCIPSKALLHSSHMYHEAKHAFANHGVKFSSVEVDLPAMMAQKDKAVSNLTKGIEGLFKKNKVN
YVKGYGKFVSPSEVSVDTTEGGNTVVKGKHIIIATGSDVKSLPGVTIDEKKVVSSTGALALTEIPKRLVVIGAGYIGLEMGSVWGRLGTEITVVEFASEIVPTMDAEVRKQFQRSLEKQGLKFKLKTKVVGVDTSGDGVKLTLEPAAG
GDQTTLEADVVLVSAGRTPFTAGLGLDKIGVETDKIGRILVNERFATNVSGVYAIGDVIPGPMLAHKAEEDGVA**C**VEYIAGKVGHVDYDKVPGVVYTMPEVASVGKTEEQVKELGVEYRVGKFPFMANSRAKAIDNAEGLVKILA
EKETDKILGVHIMAPNAGELIHEAAIALQYDASSEDIARV**C**HAHPTMSEAVKEAAMATYDKPIHI*

Threshold	Position	Peptide	Score	Cutoff	Cluster
High	365	AEEDGVACVEYIAGK	2.565	2.443	Cluster B
Low	476	SEDIARVCHAHPTMS	1.224	0.797	Cluster A

Figure S2. Predicted S-Nitrosylation sites in the protein sequence of FLbR-2.

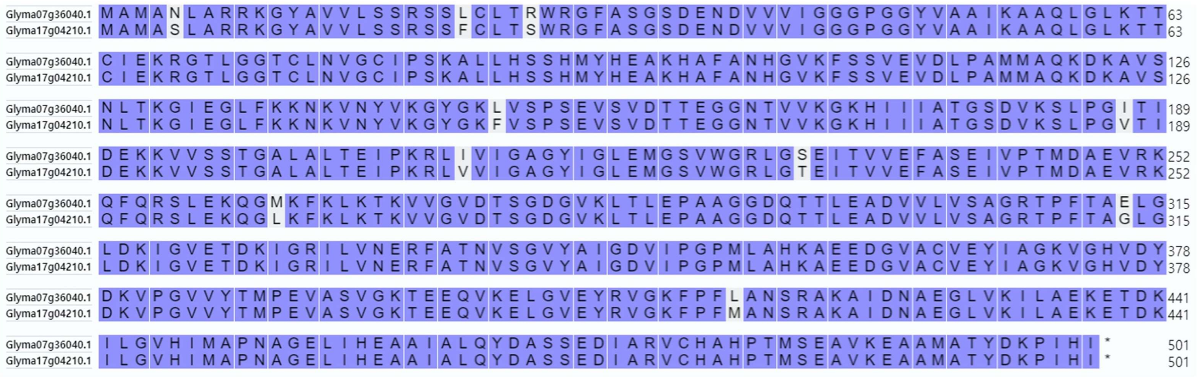

Figure S3. Peptide sequence alignment of two FLbR genes.
